# Approximation to Second Order N‑Electron Valence State Perturbation Theory: Limiting the Wave Function within Singles

**DOI:** 10.1021/acs.jctc.5c00582

**Published:** 2025-05-28

**Authors:** Yang Guo, Katarzyna Pernal

**Affiliations:** † School of Chemistry and Chemical Engineering, 12589 Shandong University, Qingdao 266237, Shandong, China; ‡ Institute of Physics, Lodz University of Technology, ul. Wolczanska 217/221, Lodz 93-005, Poland

## Abstract

Inspired by the linearized adiabatic connection (AC0) theory, an approximation to second-order N-electron valence state perturbation theory (NEVPT2) has been developed, termed NEVPT within singles (NEVPTS). This approach utilizes amplitudes derived from approximate single-excitation wave functions, requiring only 3rd-order reduced density matrices (RDMs). Consequently, it avoids the computational bottleneck associated with the construction of 4th-order RDMs in NEVPT2. The NEVPTS method demonstrates comparable performance to NEVPT2 in describing potential energy curves for diatomic molecules and singlet–triplet gaps in biradicals, while achieving superior accuracy to AC0 in these applications. For excitation energies of organic molecules, NEVPTS is less accurate than NEVPT2. The overall performance and computational costs of the NEVPTS method lie between those of NEVPT2 and AC0.

## Graphical abstract

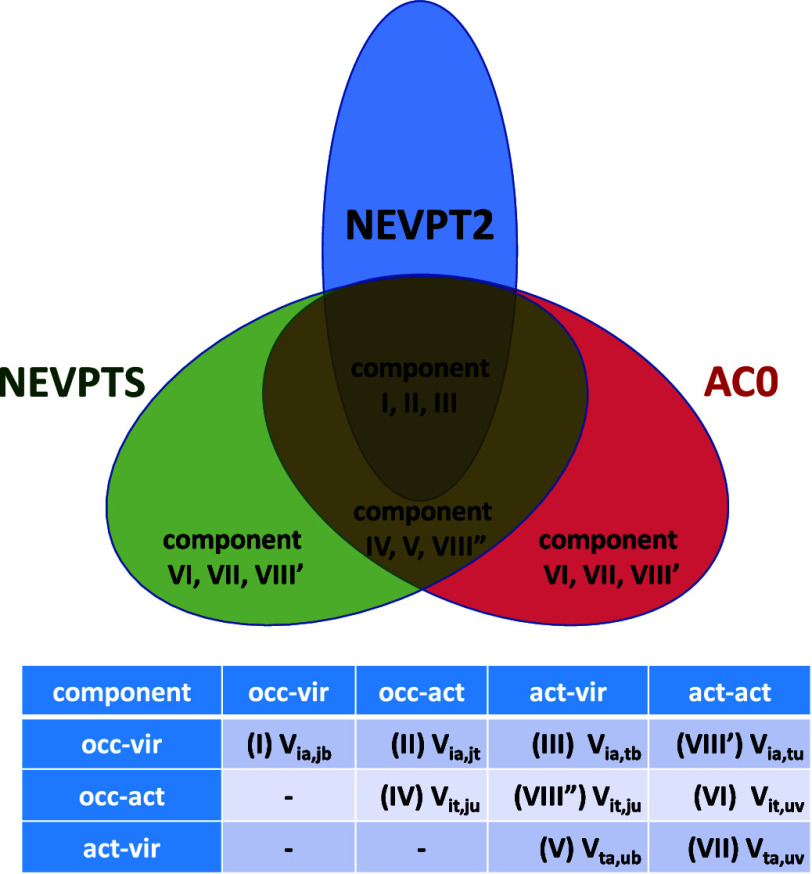

## 1 Introduction

The complete active space self-consistent field (CASSCF) method, introduced by Roos and collaborators, is a foundational multireference (MR) approach in quantum chemistry.^1,2^ Its widespread application is due to its ability to capture static correlation of strongly correlated systems. In CASSCF calculations, it is necessary to define active spaces, consisting of *m* active electrons and *n* active molecular orbitals (MOs), denoted as CAS­(*m*,*n*). Within the active space, full configuration interaction (FCI) calculations are performed, making the size of the active space a bottleneck in CASSCF applications. Typical CASSCF or CASCI algorithms utilizing conventional computers can address systems with less than 20 active electrons and MOs.^3^ To treat systems with larger active spaces, truncated CI calculations have to be performed within the active space. These methods, known as multiconfigurational SCF (MCSCF), were developed earlier than CASSCF.^4–8^ The popular MCSCF methods include restricted active space SCF (RASSCF),^9,10^ generalized active space (GAS) SCF,^11,12^ occupation-restricted-multiple-active-space (ORMAS),^13^ or selected CI SCF methods developed in the past decades.^14–26^ Alternatively, the density matrix renormalization group (DMRG) SCF methods,^27–33^ and FCI quantum Monte Carlo (FCIQMC) SCF methods^34–38^ have been reported, aiming to address systems with large active spaces as well.

To accurately simulate strongly correlated systems, it is essential to further account for dynamic correlation. Various MR dynamic correlation methods based on CASSCF or MCSCF references have been developed, including MR perturbation theories (MRPT), MR configuration interaction methods (MRCI), as well as MR coupled cluster approaches (MRCC). These MR dynamic correlation methods have proven effective in describing a variety of complex systems, including bond-breaking processes, the spin states of transition metal complexes, and excited states. Comprehensive reviews are available that provide detailed insights into these MR methodologies.^39–43^ Due to the relatively low computational costs, MRPT methods have gained greater popularity compared to MRCI and MRCC. Among the widely available MRPT methods, complete active space second-order perturbation theory (CASPT2)^44,45^ based on the generalized Fock Hamiltonian and second-order N-electron valence state perturbation theory (NEVPT2)^46,47^ based on the Dyall Hamiltonian^48^ have been extensively calibrated and widely utilized. Note that there are different variants of NEVPT2 depending on the type of wave functions: strongly contracted, internally contracted (IC), and uncontracted NEVPT2. In the present work, NEVPT2 represents IC NEVPT2, unless otherwise specified. Other MRPT2 methods have also been reported, including various MRPT2 methods by Surján and co-workers,^49–51^ generalized Van Vleck perturbation theory by Hoffmann,^52,53^ static-dynamic-static perturbation theory by Liu,^54,55^ and DSRG-MRPT2 by Evangelista,^56,57^ among others.^58,59^ Significant progress has been made in enhancing the capabilities of MRPT2. Analytical gradients of various MRPT2 methods have been developed, enabling geometry optimizations.^60–63^ The incorporation of explicit correlation techniques has improved the accuracy of MRPT2 methods to the basis set limit.^64–67^ Furthermore, the introduction of pair natural orbitals (PNOs) to MRPT has significantly increased computational efficiency, allowing the study of larger systems at the MRPT2 levels.^68–72^

In addition to advancements in analytical gradients, explicitly correlated methods, and PNO techniques, one of the most popular areas of research is the development of MRPT2 methods to treat large active spaces.^73–87^ In the context of internally contracted MRPT2 methods,^88,89^ at least 3rd-order reduced density matrices (RDMs) must be constructed.^56^ In CASPT2 or NEVPT2, 4th-order RDMs are required. To achieve a robust NEVPT2 theory with an approximate CASSCF reference, Neese and co-workers found that even 5th-order RDMs must also be constructed.^90,91^ To avoid high-order RDMs in MRPT2, Pernal and co-workers introduced the linearized adiabatic connection (AC0) theory for CASSCF or geminal reference, in which only 1st- and 2nd-order RDMs are required.^92,93^ Some benchmark studies of AC0 have demonstrated its ability to produce potential energy curves (PEC) for ground states with accuracy comparable to CASPT2 and NEVPT2.^94–97^ However, the AC0 approximation overestimates the excitation energies of organic molecules. Thus, the AC0D^98^ and ff-AC0 ^99^ methods are proposed by some of us to improve the accuracy of AC0 in describing the electronic structure of excited states. The performance of ff-AC0, which combines the advantages of particle-hole and particle–particle AC0, is comparable to that of NEVPT2. Recently, we have also elucidated the relationship between AC0 and NEVPT2.^100^ The correlation energy of AC0 can be decomposed into eight subspaces, similar to NEVPT2. We have demonstrated that for the three subspaces that involve no active MO and those with only one active MO, the contributions to the correlation energy in AC0 and NEVPT2 are identical.

In this work, based on our study of the relationship between NEVPT2 and AC0, a new MRPT2 method is developed as an approximation to NEVPT2. The amplitudes of the proposed method are derived from the approximate single excitation wave functions, while the energy expressions are similar to NEVPT2. Consequently, the new method is named as NEVPT within singles (NEVPTS). Following the introduction to the theory, the performance of NEVPTS is assessed through the PEC of diatomic molecules, singlet–triplet gaps of biradicals, and excitation energies of organic molecules. For dynamic correlation calculations, the NEVPTS method should be able to treat systems with larger active space references than NEVPT2 does, due to its independence on higher than 3rd-order density matrices.

## 2 Theory

Throughout the work, the following conventions are used, {*i*, *j*, *k*, *l*,···} represent doubly occupied (labeled as *occ*) orbitals, {*t*, *u*, *v*, *w*,···} denote active (labeled as *act*) orbitals, {*a*, *b*, *c*, *d*,···} refer to virtual (labeled as *vir*) MOs, and {*p*, *q*, *r*, *s*} stand for unspecified orbitals. The superscripts PTS and PT2 represent NEVPTS and NEVPT2, respectively.

### 2.1 Recapitulation of AC0 and NEVPT2 Theory

Since the NEVPTS method is inspired by AC0 and NEVPT2, it is essential to provide a brief introduction to the foundations of the two methods.

In AC0, the 0th-order extended random phase approximation (ERPA) equation^101^ must be solved^93^

1 $$\left(\begin{array}{ll} A^{0} & B^{0}\\ B^{0} & A^{0} \end{array}\right)\left(\begin{array}{l} X_{\mu }\\ Y_{\mu } \end{array}\right)=w_{\mu }^{0}\left(\begin{array}{cc} S & 0\\ 0 & -S \end{array}\right)\left(\begin{array}{l} X_{\mu }\\ Y_{\mu } \end{array}\right)$$

where, as a result of assuming the killer condition and the commutator metric^102^ in the equation of motion underlying ERPA, the matrices ***A***, ***B*** and ***S*** are defined as

2 $$A_{pqrs}^{\alpha }=B_{pqsr}^{\alpha }=\frac{1}{2}\left\langle \Psi_{0}\left|\left[{Ê}_{q}^{p},\left[{Ĥ}^{\alpha },{Ê}_{r}^{s}\right]\right]\right|\Psi_{0}\right\rangle ,,\left(\alpha =0,1\right)$$

3 $$S_{pqrs}=\frac{1}{2}\left\langle \Psi_{0}\left|\left[{Ê}_{q}^{p},{Ê}_{r}^{s}\right]\right|\Psi_{0}\right\rangle$$

4 $${Ê}_{q}^{p}=a_{p_{\alpha }}^{†}a_{q_{\alpha }}+a_{p_{\beta }}^{†}a_{q_{\beta }}$$

and the main matrices involve double commutators. The ${Ĥ}^{\alpha }$ in eq 2 is the Dyall Hamiltonian^48^ $\left({Ĥ}^{Dyall}\right)$ when α = 0, and the exact Hamiltonian (Ĥ) when α = 1. |Ψ_0_⟩ denotes the CASSCF or CASCI reference wave function with energy *E* ^CAS^. Due to the usage of ${Ĥ}^{Dyall}$ , the 0th-order ERPA equation (eq 1) can be structured in a block diagonal form, consisting of four distinct blocks, occ-vir, occ-act, act-vir, as well as act-act blocks.^100^ For example, only active indices are engaged in the act-act block of the 0th-order ERPA equation

$$\left(\begin{array}{ll} A^{0,act‐act} & B^{0,act‐act}\\ B^{0,act‐act} & A^{0,act‐act} \end{array}\right)\left(\begin{array}{l} X_{\mu }^{act‐act}\\ Y_{\mu }^{act‐act} \end{array}\right)=w_{\mu }^{act‐act}\left(\begin{array}{cc} S^{act‐act} & 0\\ 0 & -S^{act‐act} \end{array}\right)\left(\begin{array}{l} X_{\mu }^{act‐act}\\ Y_{\mu }^{act‐act} \end{array}\right)$$ 5

where

$$A_{tuvw}^{0,act‐act}=B_{tuwv}^{0,act‐act}=\frac{1}{2}\left\langle \Psi_{0}\left|\left[{Ê}_{u}^{t},\left[{Ĥ}^{0},{Ê}_{v}^{w}\right]\right]\right|\Psi_{0}\right\rangle ,,S_{tuvw}^{act‐act}=\frac{1}{2}\left\langle \Psi_{0}\left|\left[{Ê}_{u}^{t},{Ê}_{v}^{w}\right]\right|\Psi_{0}\right\rangle$$ 6

The detailed ERPA equations of the remaining three blocks were documented elsewhere.^100^ Utilizing the eigenvalues and eigenvectors (eigenpairs) obtained from these 0th-order ERPA equations, the correlation energy of AC0 can be computed as follows^93^

$$E^{AC0}=\frac{1}{2}\underset{\lambda \mu }{\sum }\left[\underset{pqrs}{\sum }\left(pq|rs\right){\left[S{Z_{\lambda }}^{-}\right]}_{pq}{\left[S{Z_{\mu }}^{-}\right]}_{rs}\right]\frac{{Z_{\lambda }}^{+}\left(A^{1}+B^{1}\right){Z_{\mu }}^{+}-{Z_{\lambda }}^{-}\left(A^{1}-B^{1}\right){Z_{\mu }}^{-}}{w_{\lambda }+w_{\mu }},\left({Z_{\lambda }}^{+}=X_{\lambda }+Y_{\lambda },,{Z_{\lambda }}^{-}=X_{\lambda }-Y_{\lambda }\right)$$ 7

According to the blocks to which the λ and μ indices belong, the AC0 correlation energies can be categorized into nine distinct components (Table 1).^100^

**Table 1:**
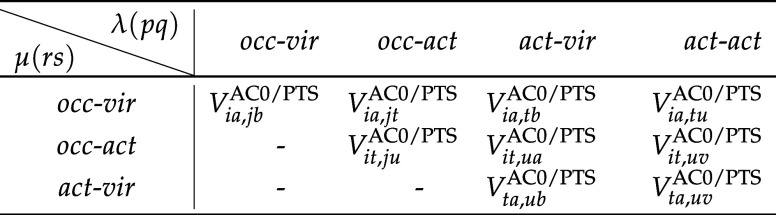
Decomposition of AC0 Correlation Energy (eq 7) into Nine Different Components Depending on the λ and μ^a^

a The correlation energy within the NEVPTS framework (eq 23) follows the same decomposition pattern.

In the NEVPT2 theory,^47,89^ the 1st-order wave function reads

8 $$|\Psi_{1}\rangle =\frac{1}{2}\sum_{pqrs}T_{pqrs}^{PT2}{Ê}_{p}^{q}{Ê}_{r}^{s}|\Psi_{0}\rangle$$

In eq 8, the IC single and double excitations in the first order interaction space (FOIS) could be divided into eight distinct subspaces depending on the indexes (Table 2).^47^ Note that the IC singles, ${Ê}_{i}^{t}|\Psi_{0}\rangle$ , ${Ê}_{i}^{a}|\Psi_{0}\rangle$ , and ${Ê}_{t}^{a}|\Psi_{0}\rangle$ , are implicitly considered in the VI, VII, VIII′ components, respectively.^103^ For example, in the VII component, when u = v, the expression ${Ê}_{u}^{v}{Ê}_{t}^{a}|\Psi_{0}\rangle$ simplifies to ${Ê}_{t}^{a}|\Psi_{0}\rangle$ . In fact, these single excitations are combined with the doubles to form an orthonormalized IC basis. The amplitudes *T*_pqrs_^PT2^ are determined by solving a set of perturbative equations within each subspace.^47,68^ Using the 1st-order wave function, the NEVPT2 correlation energy is computed as

9 $$E^{PT2}=\left\langle \Psi_{0}\left|Ĥ-{Ĥ}^{Dyall}\right|\Psi_{1}\right\rangle$$

10 $$=\left\langle \Psi_{0}\left|Ĥ\right|\Psi_{1}\right\rangle -\left\langle \Psi_{0}\left|{Ĥ}^{Dyall}\right|\Psi_{1}\right\rangle$$

11 $$=\left\langle \Psi_{0}\left|Ĥ\right|\Psi_{1}\right\rangle$$

12 $$=\sum_{pqrs}T_{pqrs}^{PT2}\left\langle \Psi_{0}\left|Ĥ\right|\frac{1}{2}{Ê}_{p}^{q}{Ê}_{r}^{s}\Psi_{0}\right\rangle$$

13 $$=\sum_{pqrs}T_{pqrs}^{PT2}N_{pqrs}$$

**Table 2:** Internally Contracted First-Order Interaction Space (FOIS) and the Relationship between Subspaces in NEVPT2 and Components in PTS and AC0^a^

No.	FOIS	PT2 subspace	AC0/PTS component
I	${Ê}_{j}^{b}{Ê}_{i}^{a}|\Psi_{0}\rangle$	*V*_*ijab*_^PT2^	*V*_*ia*,*jb*_^AC0^ = *V*_*ia*,*jb*_^PTS^
II	${Ê}_{j}^{t}{Ê}_{i}^{a}|\Psi_{0}\rangle$	*V*_*ija*_^PT2^	*V*_*ia*,*jt*_^AC0^ = *V*_*ia*,*jt*_^PTS^
III	${Ê}_{t}^{b}{Ê}_{i}^{a}|\Psi_{0}\rangle$	*V*_*iab*_^PT2^	*V*_*ia*,*tb*_^AC0^ = *V*_*ia*,*tb*_^PTS^
IV	${Ê}_{j}^{u}{Ê}_{i}^{t}|\Psi_{0}\rangle$	*V*_*ij*_^PT2^	*V*_*it*,*ju*_^AC0^ = *V*_*it*,*ju*_^PTS^
V	${Ê}_{u}^{b}{Ê}_{t}^{a}|\Psi_{0}\rangle$	*V*_*ab*_^PT2^	*V*_*ta*,*ub*_^AC0^ = *V*_*ta*,*ub*_^PTS^
VI	${Ê}_{u}^{v}{Ê}_{i}^{t}|\Psi_{0}\rangle$	*V*_*i*_^PT2^	*V*_*it*,*uv*_^AC0/PTS^
VII	${Ê}_{u}^{v}{Ê}_{t}^{a}|\Psi_{0}\rangle$	*V*_*a*_^PT2^	*V*_*ta*,*uv*_^AC0/PTS^
VIII′	${Ê}_{t}^{u}{Ê}_{i}^{a}|\Psi_{0}\rangle$	*V*_*ia*_^PT2^	*V*_*ia*,*tu*_^AC0/PTS^
VIII″	${Ê}_{u}^{a}{Ê}_{i}^{t}|\Psi_{0}\rangle$		*V*_*it*,*ua*_^AC0^ = *V*_*it*,*ua*_^PTS^

a For I–VII, there is a one-to-one correspondence between PTS/AC0 components and NEVPT2 subspaces. However, the remaining two components, VIII′ and VIII″ in PTS/AC0, are treated as one single subspace in NEVPT2. This subspace in NEVPT2 is denoted as VIII. Components I–V, VIII″ are identical in PTS and AC0.

In eq 10, the second term vanishes because |Ψ_0_⟩ is an eigenvector of the Dyall Hamiltonian, ${Ĥ}^{Dyall}|\Psi_{0}\rangle =E^{CAS}|\Psi_{0}\rangle$ . Recently, we established a correspondence between the subspaces in NEVPT2 and the components in AC0,^100^ as summarized in Table 2. The I–VII subspaces in NEVPT2 correspond to the seven components of AC0, while the VIII′ and VIII″ components of AC0 correspond to a single subspace of NEVPT2.^100^ Furthermore, by comparing the explicit correlation energy expressions of AC0 and NEVPT2, we found that the I–III components of AC0 are identical to the corresponding subspaces of NEVPT2.^100^

### 2.2 NEVPTS

In AC0, the ***B*** ^0^ and ***Y***_μ_ in eq 1 are zero for occ-vir, occ-act, act-vir blocks.^100^ Thus, the correlation energies for the six components, I–V, and VIII″ could be simplified

14 $$E_{I-V,{VIII}^{\prime \prime }}^{AC0}=\sum_{\lambda \mu }\sum_{pqrs}{\left[SX_{\lambda }\right]}_{pq}\left(pq|rs\right){\left[SX_{\mu }\right]}_{rs}\frac{X_{\lambda }B^{1}X_{\mu }}{w_{\lambda }+w_{\mu }}$$

Notice that the general indices pqrs are assumed to be constrained to one of the components, according to Table 2. Equation 14 can be further simplified by expanding the double commutator in ***B*** ^1^. It turns out that the final expressions of ***B*** ^1^ in the six components are identical to those in the NEVPT2 energy expression, eq 13

15 $$B_{pqrs}^{1}=\left\langle \Psi_{0}\left|Ĥ\right|\frac{1}{2}{Ê}_{p}^{q}{Ê}_{r}^{s}\Psi_{0}\right\rangle =N_{pqrs}$$

Thus, the AC0 correlation energy expression for the six components, I–V and VIII″, listed in Table 2 can be written as

$$E_{I-V,{VIII}^{\prime \prime }}^{AC0}=\underset{pqrs}{\sum }\left(pq|rs\right)T_{pqrs}^{PTS},,\left(T_{pqrs}^{PTS}=\underset{\lambda \mu }{\sum }{\left[SX_{\lambda }\right]}_{pq}\frac{X_{\lambda }NX_{\mu }}{w_{\lambda }+w_{\mu }}{\left[SX_{\mu }\right]}_{rs}\right)$$ 16

For the I–III components, besides the numerators, the integrals (pq|rs) as well as the denominators in *T*_pqrs_^PTS^ are also identical to those in NEVPT2. In contrast, the integrals and denominators in the IV–V and VIII″ components are different in AC0 and NEVPT2.^100^ Equation 16 could also be derived using configuration interaction singles (CIS) wave functions. Thus, the ERPA solutions for the I–V and VIII″ components are equivalent using ERPA and CIS. Inspired by this equation, the single excitation wave functions can also be employed as a substitute for the act-act block of ERPA (eq 5) to compute the correlation energies of the remaining three components. The single excitation wave function within the active space is computed as follows

$${Ã}^{act‐act}{\mathrm{X̃}}_{\lambda }^{act‐act}={\mathrm{w̃}}_{\lambda }^{act‐act}{\mathrm{S̃}}^{act‐act}{\mathrm{X̃}}_{\lambda }^{act‐act}$$ 17

$${\mathrm{S̃}}_{tuvw}^{act‐act}=\frac{1}{2}\left\langle \Psi_{0}\left|{Ê}_{u}^{t}{Ê}_{w}^{v}\right|\Psi_{0}\right\rangle$$ 18

$${Ã}_{tuwv}^{act‐act}=\frac{1}{2}\left\langle \Psi_{0}\left|{Ê}_{u}^{t}\left[{Ĥ}^{Dyall},{Ê}_{w}^{v}\right]\right|\Psi_{0}\right\rangle =\frac{1}{2}\left\langle \Psi_{0}\left|{Ê}_{u}^{t}{Ĥ}^{Dyall}{Ê}_{w}^{v}\right|\Psi_{0}\right\rangle -{\mathrm{S̃}}_{tuvw}^{act‐act}E^{CAS}$$ 19

The matrices ***A* ~** and ***S* ~** are identical to those in the *V*_*ia*_^PT2^ subspace of NEVPT2, as detailed elsewhere.^47^ Notice that compared with the ERPA equations based on the commutator metric, they are based on a simple metric^102^ and the matrices involve one commutator less than their ERPA counterparts (eq 6). With the eigenpairs obtained from eq 17, the correlation energy of the remaining three components, VI–VII, and VIII′ are also computed using eq 16.

The above new approach is designated as NEVPT singles (NEVPTS). In NEVPTS, the approximate single excitation wave function of reference is computed

20 $$Ã{\mathrm{X̃}}_{\lambda }={\mathrm{w̃}}_{\lambda }\mathrm{S̃}{\mathrm{X̃}}_{\lambda }$$

where ***A* ~**, ***S* ~** are defined as

21 $${\mathrm{S̃}}_{pqrs}=\frac{1}{2}\left\langle \Psi_{0}\left|{Ê}_{q}^{p}{Ê}_{r}^{s}\right|\Psi_{0}\right\rangle$$

22 $${Ã}_{pqrs}=\frac{1}{2}\left\langle \Psi_{0}\left|{Ê}_{q}^{p}\left[{Ĥ}^{Dyall},{Ê}_{r}^{s}\right]\right|\Psi_{0}\right\rangle =\frac{1}{2}\left\langle \Psi_{0}\left|{Ê}_{q}^{p}{Ĥ}^{Dyall}{Ê}_{r}^{s}\right|\Psi_{0}\right\rangle -{\mathrm{S̃}}_{pqrs}E^{CAS}$$

The final correlation energies are computed as follows

23 $$E^{PTS}=\sum_{pqrs}\left(pq|rs\right)\sum_{\lambda \mu }\left[{\left[\mathrm{S̃}{\mathrm{X̃}}_{\lambda }\right]}_{pq}\frac{{\mathrm{X̃}}_{\lambda }N{\mathrm{X̃}}_{\mu }}{{\mathrm{w̃}}_{\lambda }+{\mathrm{w̃}}_{\mu }}{\left[\mathrm{S̃}{\mathrm{X̃}}_{\mu }\right]}_{rs}\right]=\sum_{pqrs}\left(pq|rs\right)T_{pqrs}^{PTS}$$

Note that, in contrast to eq 18–19, the pqrs in eq 21–22 are general indices. Similar to the ERPA equation employed in AC0, eq 20 also exhibits a block diagonal structure, comprising four blocks. Thus, the correlation energy of NEVPTS (eq 23) can be similarly decomposed into nine components, Table 1. Components I–V and VIII″ in NEVPTS are identical to those in AC0. For the remaining three components that involve act-act single excitations, the correlation energies of NEVPTS are derived from eigenpairs of eq 17, whereas in AC0, they are computed by the eigenpairs of the ERPA equation. The number of eigenpairs in the act-act block of NEVPTS is generally greater than in AC0 (see Appendix 2), allowing NEVPTS to recover more correlation energies associated with act-act excitations. However, the computation of ***A* ~** in NEVPTS requires the construction of 3rd-order RDMs, whereas AC0 only involves 2nd-order RDMs. Consequently, the computational costs of NEVPTS are higher than those of AC0. Nonetheless, the computational costs of NEVPTS remain lower than those of NEVPT2, as the latter requires higher than 3rd-order RDMs.

Interestingly, the NEVPTS theory is closely related to the Tamm-Dancoff-approximated^104^ AC0 theory (AC0tda), which has not been reported yet. NEVPTS can be seen as a Tamm-Dancoff approximation to AC0 based on an alternative metric. The basic theory of AC0tda and its relationship with NEVPTS are discussed in Appendix 1.

## 3 Results

The NEVPTS method has been implemented in a development version of ORCA.^105–107^ The resolution of identity (RI) approximation is employed to reduce the computational costs associated with two-electron integrals in all NEVPTS, AC0, and NEVPT2 calculations.

### 3.1 Potential Energy Curves (PECs) of Diatomic Molecules

The NEVPTS method is used to investigate the PECs of N_2_, O_2_, and Cr_2_ molecules. For the PEC of N_2_, a CAS­(10,8) active space is employed, whereas CAS­(8,6) is used for O_2_. Both molecules are computed with cc-pwCVQZ basis set^108^ without the frozen core approximation. For the PEC of Cr_2_, computations are performed at the CAS­(12,12)/cc-pVQZ level.^109^ The 1s2s2p orbitals of Cr atoms are defined as core orbitals. The absolute correlation energies of these three diatomic molecules computed by NEVPTS are compared with those obtained from NEVPT2 and AC0. It has been established that the energy expressions for the I–III components (subspaces) in the AC0, NEVPTS, and NEVPT2 methods are identical.^100^ Thus, only the remaining six components of NEVPTS or AC0 are discussed in this subsection. In the comparison, the correlation energies of the IV–VII components in NEVPTS or AC0 could be directly compared with those of the corresponding subspaces in NEVPT2. However, the summations of component VIII′ and VIII″ in NEVPTS and AC0 are associated with the results from the *V*_*ia*_^PT2^ subspace of NEVPT2. Hence, in the rest of the work, the sum of VIII′ and VIII″ components is referred to as the VIII component. Furthermore, as elucidated in the theory of NEVPTS, the correlation energies of the IV and V components are identical in both NEVPTS and AC0. Thus, a single set of results is presented for these two components.

The results for N_2_ are presented in Figure 1. As illustrated in Figure 1a, the correlation energies of the IV, VI, and VIII components in N_2_ are almost the same across all three methods, with deviations less than 0.1 mEh along the PEC. For the V component, the NEVPTS (AC0) method yields lower correlation energies compared to NEVPT2. Regarding component VII, the NEVPTS method provides more reliable results than AC0 does, using NEVPT2 results as the reference. The NEVPTS curve exhibits no bumps. Therefore, the binding energy produced by NEVPTS is more consistent with that of NEVPT2, whereas the binding energy of N_2_ is underestimated by AC0 compared to NEVPTS or NEVPT2 (Figure 1b). It is worth noting that the absolute energies delivered by NEVPTS are lower than those by AC0 (see the Supporting Information).

**Figure 1:**
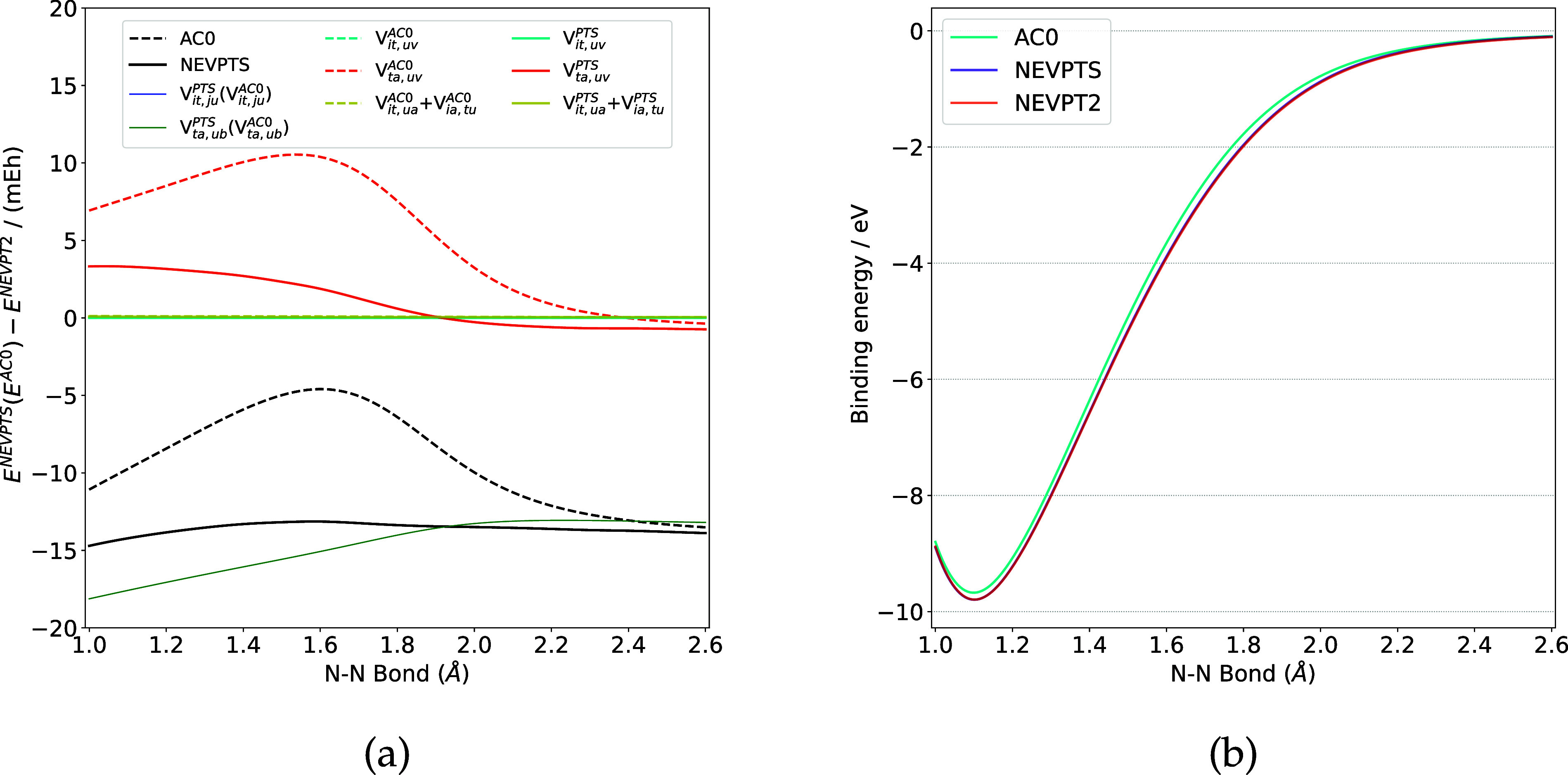
(a) Deviations (in mEh) of NEVPTS and AC0 correlation energies relative to NEVPT2 for N_2_. (b) Binding energy curves (in eV) of N_2_.

The correlation energies of the ground state (triplet state) of O_2_ are presented in Figure 2. Analogous to the findings for N_2_, the correlation energies of the V component are overestimated by NEVPTS (AC0) by approximately 15 mEh across the PEC. Conversely, the energies of the VIII component are underestimated by both NEVPTS and AC0. For the components VI and VII, the NEVPTS method produces more accurate results compared to AC0, with discrepancies from NEVPT2 being less than 1.2 mEh. Thus, the binding energy curve of O_2_ calculated by NEVPTS, as shown in 2­(b), is closer to that obtained at the NEVPT2 level.

**Figure 2:**
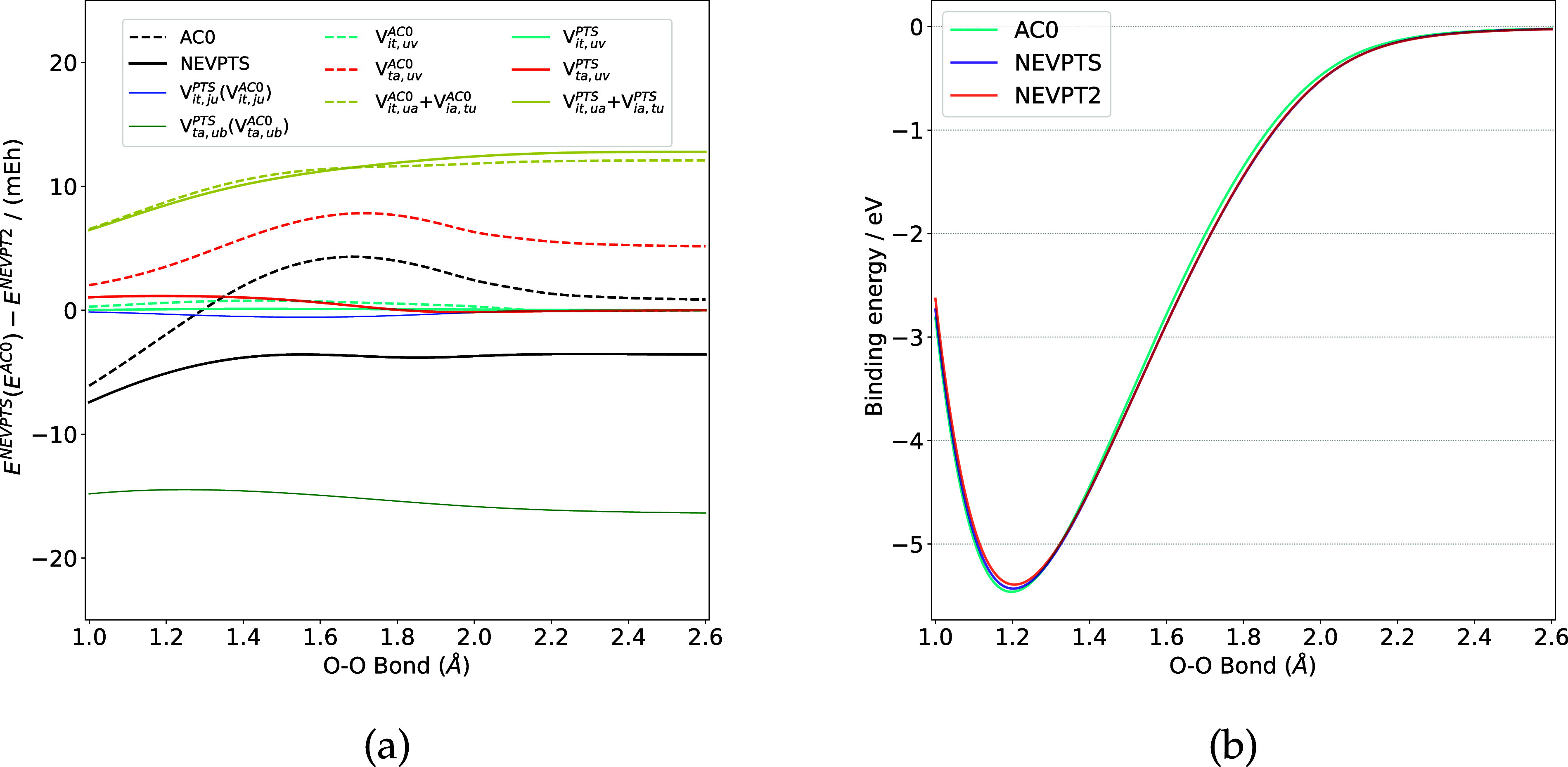
(a) Deviations (in mEh) of NEVPTS and AC0 correlation energies relative to NEVPT2 for O_2_. (b) Binding energy curves (in eV) of O_2_.

The PEC of Cr_2_ is also studied using the three methods, as shown in Figure 3. Similar to the results of N_2_ and O_2_, the correlation energies of the IV and V components are overestimated by NEVPTS and AC0. Both AC0 and NEVPTS underestimate the energy of the VIII component relative to NEVPT2. For the components VI and VII, the absolute correlation energies computed by NEVPTS are comparable to those by NEVPT2, while the AC0 method underestimates the correlation energies of the component VII by more than 20 eV at the equilibrium geometry (1.7 Å). Consequently, the binding energies (Figure 3b) delivered by NEVPTS and NEVPT2 are close to each other and more accurate than those predicted by AC0, compared to experimental results.^110^ To gain deeper insights into the satisfactory results from NEVPTS, the eigenvalues involved in the denominator of the component VII in NEVPTS and AC0 are compared with the eigenvalues of the Koopmans matrices in that of NEVPT2.^89^ In NEVPT2, the Koopmans eigenvalues are only relevant to active MOs.^89^ Thus, the virtual MO energies in the denominators of the VII component of NEVPTS and AC0 are excluded from the comparison. The remaining parts in the denominators of NEVPTS and AC0 consist of two contributions involving active indices (see Appendix 2 for more details). The sum of the two contributions is compared with the Koopmans eigenvalues in NEVPT2. Eigenvalues for the component VII of Cr_2_ at a bond length of 1.7 Å are provided in Figure 4. Surprisingly, the total number of eigenvalues in NEVPTS matches that in NEVPT2. In the low-energy region, the eigenvalues from NEVPTS are larger than those in NEVPT2, which could lead to greater denominators. Thus, the correlation energy of the VII component is slightly underestimated by NEVPTS relative to NEVPT2. However, due to the usage of the double commutator in ERPA equations, the total number of eigenvalues in AC0 is much less than that in NEVPTS. Moreover, the AC0 eigenvalues tend to shift upward relative to their NEVPTS counterparts. Both effects are likely the major cause of the correlation energy underestimation of AC0.

**Figure 3:**
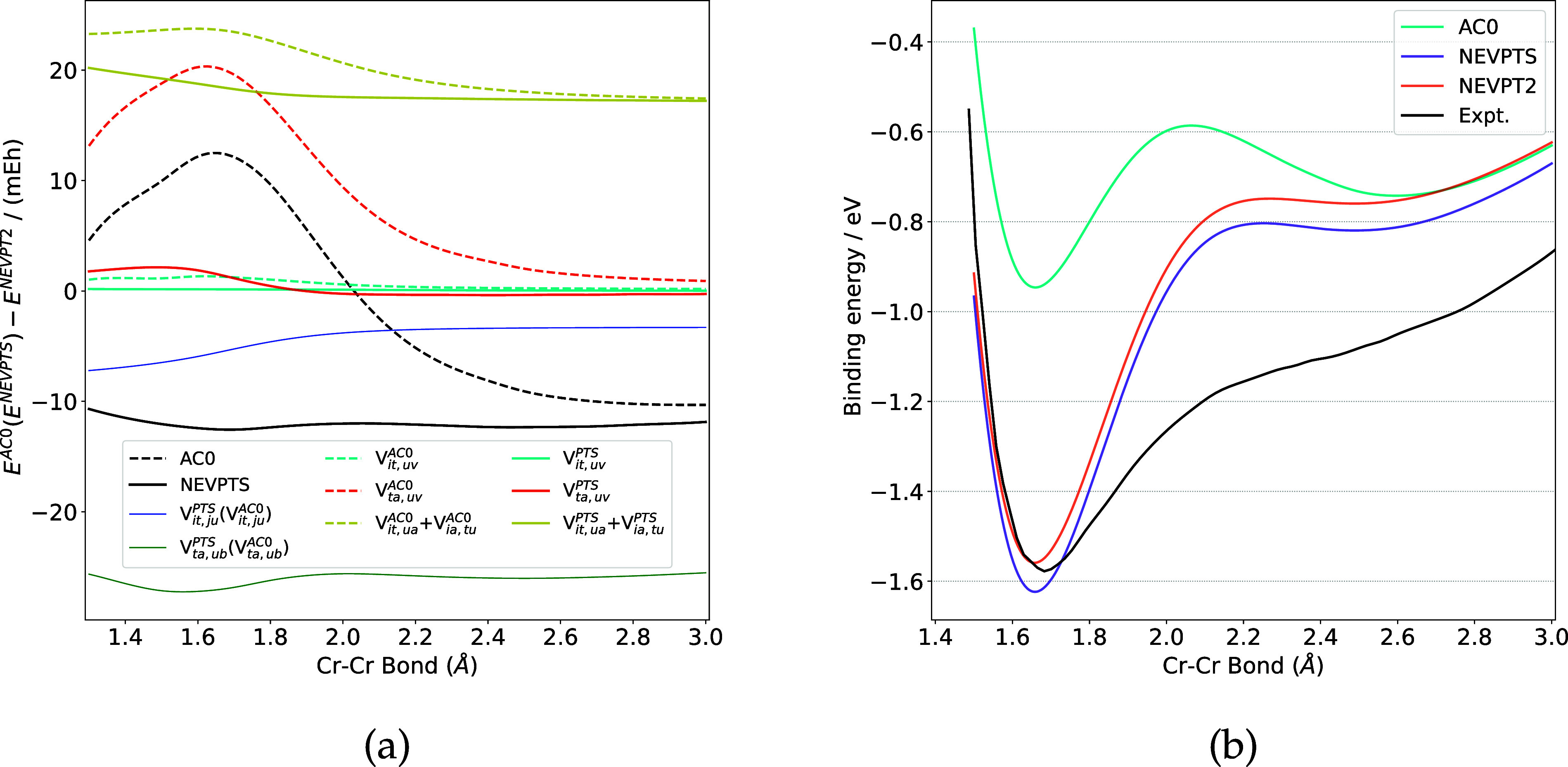
(a) The deviations (in mEh) of NEVPTS (PTS) and AC0 correlation energies with respect to NEVPT2 for Cr_2_. (b) The binding energy (in eV) curves of Cr_2_, compared with the experimental results.^110^

**Figure 4:**
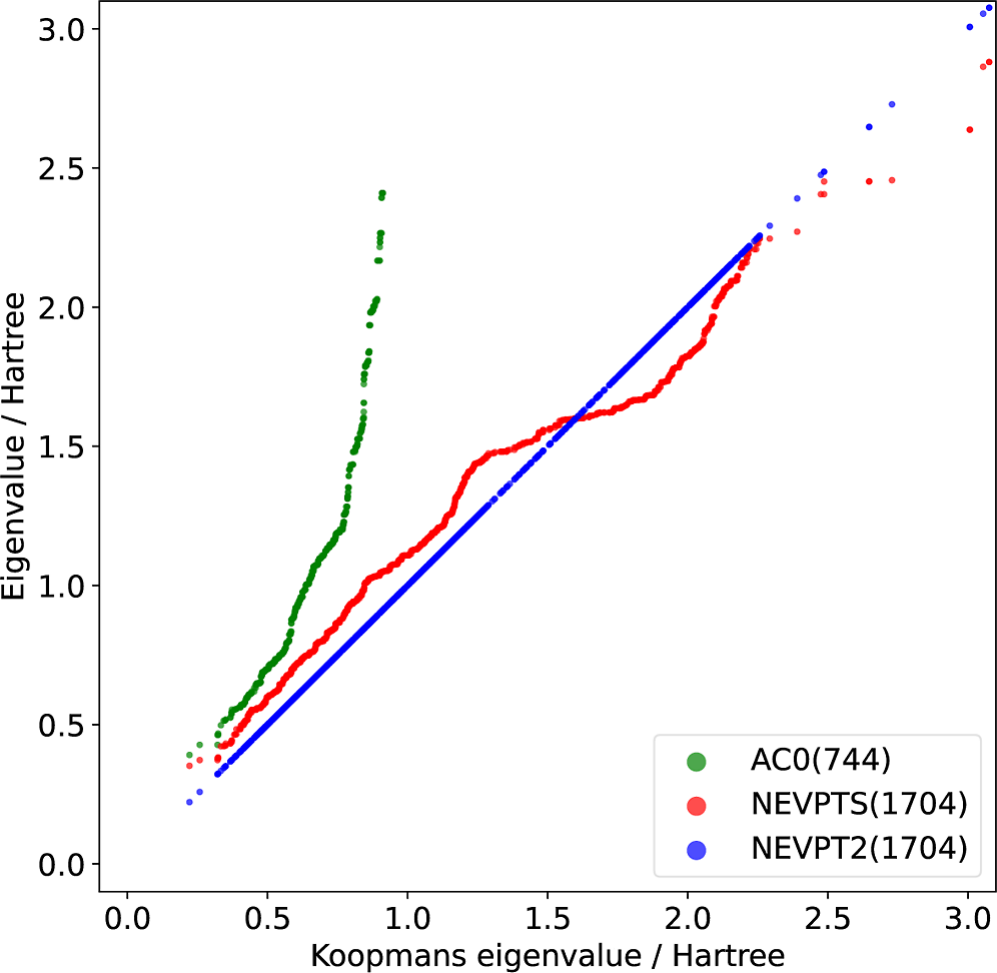
Eigenvalues (in Eh) appearing in the VII component of NEVPTS and AC0, compared with eigenvalues of Koopmans matrices in the VII subspace of NEVPT2 methods (denoted as “Koopmans eigenvalue” on the plot) for Cr_2_ at a bond length of 1.7 Å. The total number of eigenvalues in each method is given in parentheses.

### 3.2 The Singlet–Triplet Gaps of Biradicals

The singlet–triplet (S-T) gaps of biradicals, as reported by Gagliardi and co-workers,^111^ are also examined using NEVPTS, AC0, and NEVPT2 with the aug-cc-pVTZ (AVTZ) and aug-cc-pVQZ (AVQZ) basis sets. In this study, the active spaces for certain biradicals differ from those reported by Gagliardi et al.^111^ For all molecules, the active spaces encompass all π electrons and π MOs, as detailed in Table 3. When compared to the doubly electron-attached equation-of-motion (DEA-EOM) coupled-cluster (CC) results, the mean absolute deviations (MADs) of each method using AVTZ and AVQZ basis sets are nearly identical. The S-T gaps predicted by the NEVPT2 method exhibit the highest accuracy, although the MAD is sizable, amounting to 3.45 kcal/mol. The MAD of the NEVPTS method is slightly higher and equal to 3.91 kcal/mol. AC0 exhibits the largest MAD of 4.57 kcal/mol with respect to the reference values. The accuracy of NEVPTS falls between that of NEVPT2 and AC0.

**Table 3:** Deviations in the Singlet-Triplet (S-T) Gaps (in kcal/mol) of Biradicals, Computed by NEVPT2, AC0, and NEVPTS with aug-cc-pVTZ (AVTZ) and aug-cc-pVQZ (AVQZ)^a^

biradical	active space	NEVPT2		AC0		NEVPTS		EOM-CC^a^
		AVTZ	AVQZ	AVTZ	AVQZ	AVTZ	AVQZ	
C_4_H_4_	CAS(4,4)	–3.53	–3.54	–4.23	–4.24	–4.14	–4.14	4.20
C_5_H_5_^+^	CAS(5,5)	–3.51	–3.46	–5.56	–5.51	–3.37	–3.32	–13.88
C_4_H_3_NH_2_	CAS(6,5)	–3.91	–3.84	–4.63	–4.64	–4.53	–4.53	2.65
C_4_H_3_CHO	CAS(6,6)	–3.27	–3.28	–3.69	–3.71	–3.80	–3.81	3.65
C_4_H_2_NH_2_(CHO)	CAS(8,7)	–9.14	–9.16	–9.03	–9.04	–9.58	–9.59	5.68
C_4_H_2_-1,2-(CH_2_)_2_	CAS(6,6)	–0.20	–0.13	2.13	2.22	1.53	1.62	77.68
C_4_H_2_-1,3-(CH_2_)_2_	CAS(6,6)	–0.82	–0.75	–2.72	–2.64	–0.42	–0.34	–18.49
MD^b^		–3.48	–3.45	–3.47	–3.94	–3.47	–3.45	-
MAD^b^		3.48	3.45	4.57	4.57	3.91	3.91	-

a The DEA-EOM-CC/AVTZ results (EOM-CC) reported by Gagliardi and co-workers are used as the reference.^111^

b The mean deviations (MD) and mean absolute deviations (MAD) relative to EOM-CC results are given as well.

### 3.3 Excited States of Organic Molecules

The excited states of several organic molecules from Thiel’s test set^112^ are also studied using the NEVPTS method. In these calculations, the active spaces and computational procedures recommended by Thiel and colleagues are adopted. All calculations utilize the def2-TZVP basis sets with the frozen core approximation. In these calculations, the state-averaged (SA) CASSCF calculations are performed only over states with the same spin and spatial symmetries. For the excitation energies of states with the same symmetry as the ground state, the SA ground state energies serve as references. Conversely, for the excitation energies of states with different symmetries from the ground state, the state-specific CASSCF and post-CASSCF results of the ground state are utilized as references. The excitation energies computed by NEVPTS, AC0, and NEVPT2 are presented in Table 4. The CC3 results reported in ref 112 serve as a reference. The NEVPT2 method yields the most accurate excitation energies for these organic molecules, with a MAD of 0.29 eV. However, the performance of NEVPTS and AC0 is unsatisfactory. The MAD of NEVPTS is 0.73 eV, which is even greater than that of AC0.

**Table 4:** Excitation Energies (in eV) of Selected Organic Molecules, Computed by AC0, NEVPTS, and NEVPT2 Relative to CC3 Results

molecule	state	AC0	NEVPTS	NEVPT2	CC3
octatetraene CAS(8,8)	2^1^ *A*_*g*_	–0.05	0.06	–0.25	4.97
	3^1^ *A*_*g*_	0.59	0.53	0.19	6.50
	4^1^ *A*_*g*_	1.27	1.30	0.91	6.81
	1^1^ *B*_*u*_	0.04	0.14	–0.23	6.06
	2^1^ *B*_*u*_	0.26	–1.89	–1.14	4.94
	3^1^ *B*_*u*_	0.90	0.86	0.44	7.91
	1^3^ *A*_*g*_	0.17	0.12	0.05	3.67
	1^3^ *B*_*u*_	0.09	0.06	0.01	2.30
benzene CAS(6,6)	1^1^ *E*_2*g*_	0.21	0.10	–0.02	8.43
	1^1^ *B*_1*u*_	0.60	–0.67	–0.49	6.68
	1^1^ *B*_2*u*_	0.33	0.78	0.16	5.07
	1^1^ *E*_1*u*_	1.35	–1.52	–0.79	7.45
	1^3^ *E*_2*g*_	0.27	0.25	0.12	7.49
	1^3^ *B*_1*u*_	0.25	0.39	0.21	4.12
	1^3^ *B*_2*u*_	–0.87	–1.14	–1.13	6.04
	1^3^ *E*_1*u*_	0.20	0.07	0.00	4.90
furan CAS(6,5)	2^1^ *A*_1_	0.56	1.49	0.11	6.62
	3^1^ *A*_1_	2.23	1.26	–0.30	8.53
	1^1^ *B*_2_	0.94	–2.06	–0.36	6.60
	1^3^ *A*_1_	0.24	0.23	0.15	5.48
	1^3^ *B*_2_	0.13	0.23	0.16	4.17
pyrrole CAS(6,5)	2^1^ *A*_1_	0.64	1.08	0.15	6.40
	3^1^ *A*_1_	2.02	0.81	–0.08	8.17
	1^1^ *B*_2_	0.73	–1.14	–0.06	6.71
	1^3^ *A*_1_	0.26	0.21	0.15	5.51
	1^3^ *B*_2_	0.19	0.32	0.26	4.48
pyrazine CAS(10,8)	2^1^ *A*_*g*_	0.53	0.51	0.15	8.69
	1^1^ *A*_*u*_	0.31	1.16	–0.22	5.05
	1^1^ *B*_1*g*_	0.62	1.40	–0.08	6.75
	1^1^ *B*_1*u*_	1.08	–0.73	–0.55	7.07
	2^1^ *B*_1*u*_	1.74	–0.78	–0.56	8.06
	1^1^ *B*_2*g*_	0.45	0.08	0.05	5.74
	1^1^ *B*_2*u*_	0.44	0.28	0.27	5.02
	2^1^ *B*_2*u*_	1.54	–0.99	–0.79	8.05
	1^1^ *B*_3*g*_	0.14	0.04	–0.08	8.77
	1^1^ *B*_3*u*_	0.14	–0.08	–0.14	4.24
pyridazine CAS(10,8)	2^1^ *A*_1_	0.42	1.44	0.26	5.22
	3^1^ *A*_1_	1.40	2.83	1.13	7.82
	1^1^ *A*_2_	0.33	0.00	0.01	4.49
	2^1^ *A*_2_	0.75	0.14	0.08	5.74
	1^1^ *B*_1_	0.24	–0.76	–0.12	3.92
	2^1^ *B*_1_	0.87	0.88	0.18	6.41
	1^1^ *B*_2_	1.03	–0.03	0.44	6.93
	2^1^ *B*_2_	0.97	0.59	–0.20	7.55
s-triazine CAS(12,9)	2^1^ *A*_1_	1.20	–2.67	–0.48	7.41
	3^1^ *A*_1_	0.16	1.33	–0.35	9.44
	4^1^ *A*_1_	2.20	–1.00	–0.22	8.04
	1^1^ *A*_2_	0.50	0.05	–0.03	4.81
	2^1^ *A*_2_	0.76	–0.54	–0.24	4.78
	3^1^ *A*_2_	0.73	0.33	0.11	7.80
	1^1^ *B*_1_	0.29	0.28	0.21	5.71
	1^1^ *B*_2_	0.20	0.10	0.08	4.76
	MD	0.63	0.11	–0.06	-
	MAD	0.66	0.73	0.29	-

To further understand the performance of NEVPTS, the relative energies of excitation energies (ΔΔ*E*) from each component relative to those of NEVPT2 are given in Table 5. The ΔΔ*E* results of AC0 are also given for comparison. The ΔΔ*E* of IV and V components are identical in NEVPTS and AC0 theory, with small MADs of 0.00 and 0.04 eV, respectively. For the ΔΔ*E* of component VI, due to their small absolute contributions, both AC0 and NEVPTS produce results as accurate as NEVPT2. In contrast, the ΔΔ*E* of VII and VIII components are larger. Using the NEVPT2 results as references, the AC0 method produces slightly better excitation energy contributions of the VII component, while NEVPTS delivers more accurate results for the VIII component with a MAD of ΔΔ*E* of 0.26 eV. Although the excitation energies produced by AC0 are slightly better than those of NEVPTS compared to CC3 reference (Table 4), the MAD of NEVPTS is 0.16 eV lower than that of AC0 using the NEVPT2 results as reference values. Thus, using the NEVPT2 results as references, NEVPTS delivers slightly more accurate excitation energies than AC0 does. Nevertheless, the NEVPTS method could not predict excitation energies as accurately as NEVPT2, and its accuracy is comparable to AC0.

**Table 5:** Deviations (in eV) of Excitation Energies from the IV–VIII Components of Selected Organic Molecules Computed by AC0, NEVPTS (PTS) Relative to NEVPT2 Results

molecule	state	*V*_*it*,*ju*_^PTS/AC0^	*V*_*ta*,*ub*_^PTS/AC0^	*V*_*it*,*uv*_^AC0^	*V*_*it*,*uv*_^PTS^	*V*_*tu*,*va*_^AC0^	*V*_*tu*,*va*_^PTS^	*V*_*ia*,*tu*_^AC0^+*V*_*it*,*ua*_^AC0^	*V*_*ia*,*tu*_^PTS^ + *V*_*it*,*ua*_^PTS^	AC0	PTS
octatetraene CAS(8,8)	2^1^ *A*_*g*_	0.01	0.04	0.00	0.00	0.03	0.10	0.11	0.15	0.19	0.30
	3^1^ *A*_*g*_	0.01	0.06	0.00	0.00	0.16	0.16	0.17	0.11	0.40	0.33
	4^1^ *A*_*g*_	0.01	0.06	0.00	0.00	0.10	0.16	0.19	0.15	0.36	0.39
	1^1^ *B*_*u*_	0.01	0.05	0.00	0.00	0.07	0.31	0.14	–0.01	0.27	0.36
	2^1^ *B*_*u*_	0.00	–0.01	0.00	0.00	0.39	–0.61	1.02	–0.13	1.40	–0.75
	3^1^ *B*_*u*_	0.01	0.06	0.00	0.00	0.15	0.34	0.23	0.01	0.46	0.42
	1^3^ *A*_*g*_	0.01	0.04	0.00	0.00	0.02	0.02	0.04	0.00	0.12	0.07
	1^3^ *B*_*u*_	0.01	0.03	0.00	0.00	0.02	0.01	0.02	0.00	0.08	0.05
benzene CAS(6,6)	1^1^ *E*_2*g*_	0.01	0.04	0.00	0.00	0.02	0.02	0.16	0.05	0.23	0.13
	1^1^ *B*_1*u*_	0.00	–0.04	0.00	0.00	0.18	–0.03	0.95	–0.11	1.09	–0.17
	1^1^ *B*_2*u*_	0.01	0.03	0.00	0.00	0.02	0.57	0.12	0.01	0.17	0.62
	1^1^ *E*_1*u*_	0.00	0.00	0.00	0.00	0.45	–0.63	1.69	–0.10	2.15	–0.72
	1^3^ *E*_2*g*_	0.01	0.05	0.00	0.00	0.00	0.02	0.08	0.05	0.15	0.13
	1^3^ *B*_1*u*_	0.01	0.04	0.00	0.00	–0.02	0.11	0.01	0.03	0.03	0.18
	1^3^ *B*_2*u*_	0.01	0.04	0.00	0.00	0.05	–0.07	0.16	0.02	0.26	–0.01
	1^3^ *E*_1*u*_	0.01	0.03	0.00	0.00	0.03	0.02	0.13	0.00	0.20	0.07
furan CAS(6,5)	2^1^ *A*_1_	0.01	0.04	0.00	0.00	0.07	0.59	0.34	0.74	0.46	1.38
	3^1^ *A*_1_	0.00	0.03	0.00	0.00	0.49	0.09	2.02	1.44	2.53	1.56
	1^1^ *B*_2_	0.00	–0.02	0.00	0.00	0.30	–0.69	1.03	–0.99	1.30	–1.70
	1^3^ *A*_1_	0.01	0.04	0.00	0.00	–0.01	0.01	0.06	0.03	0.09	0.08
	1^3^ *B*_2_	0.00	0.02	0.00	0.00	–0.03	0.01	–0.03	0.03	–0.03	0.07
pyrrole CAS(6,5)	2^1^ *A*_1_	0.00	0.04	0.00	0.00	0.10	0.31	0.33	0.57	0.48	0.93
	3^1^ *A*_1_	0.00	0.03	0.00	0.00	0.47	–0.10	1.60	0.95	2.10	0.89
	1^1^ *B*_2_	0.00	–0.01	0.00	0.00	0.17	–0.43	0.63	–0.63	0.79	–1.07
	1^3^ *A*_1_	0.01	0.03	0.00	0.00	–0.01	0.00	0.08	0.02	0.11	0.06
	1^3^ *B*_2_	0.00	0.02	0.00	0.00	–0.05	0.00	–0.04	0.04	–0.07	0.06
pyrazine CAS(10,8)	2^1^ *A*_*g*_	0.01	0.03	0.00	0.00	0.12	0.01	0.23	0.31	0.39	0.36
	1^1^ *A*_*u*_	0.00	–0.01	0.04	0.13	0.24	0.77	0.26	0.48	0.54	1.38
	1^1^ *B*_1*g*_	0.00	–0.01	0.00	–0.05	0.36	0.95	0.34	0.58	0.69	1.48
	1^1^ *B*_1*u*_	–0.01	–0.05	0.00	0.00	0.38	–0.03	1.31	–0.09	1.63	–0.18
	2^1^ *B*_1*u*_	0.00	–0.01	0.00	0.00	0.67	–0.11	1.65	–0.09	2.30	–0.21
	1^1^ *B*_2*g*_	0.00	–0.05	0.01	0.00	0.27	–0.01	0.17	0.09	0.40	0.03
	1^1^ *B*_2*u*_	0.00	0.00	0.00	0.00	0.02	0.37	0.15	–0.36	0.17	0.02
	2^1^ *B*_2*u*_	0.00	–0.01	0.00	0.00	0.72	–0.47	1.62	0.28	2.34	–0.19
	1^1^ *B*_3*g*_	0.01	0.05	0.00	0.00	0.02	0.01	0.14	0.05	0.22	0.12
	1^1^ *B*_3*u*_	0.00	–0.04	0.03	0.01	0.16	0.00	0.13	0.09	0.29	0.07
pyridazine CAS(10,8)	2^1^ *A*_1_	0.00	0.03	0.00	0.00	–0.02	0.59	0.15	0.56	0.16	1.18
	3^1^ *A*_1_	0.01	0.05	0.00	0.00	0.05	0.77	0.18	0.88	0.28	1.71
	1^1^ *A*_2_	0.00	–0.03	0.04	0.01	0.17	–0.02	0.15	0.04	0.33	0.00
	2^1^ *A*_2_	0.00	–0.04	0.03	0.05	0.39	–0.09	0.29	0.14	0.67	0.06
	1^1^ *B*_1_	0.00	–0.05	0.04	0.03	0.20	–0.50	0.17	–0.13	0.35	–0.65
	2^1^ *B*_1_	0.00	–0.03	0.04	0.04	0.37	0.35	0.30	0.33	0.68	0.70
	1^1^ *B*_2_	0.00	–0.02	0.00	0.00	0.20	0.07	0.40	–0.52	0.59	–0.47
	2^1^ *B*_2_	0.00	–0.03	0.00	0.00	0.35	0.41	0.85	0.41	1.18	0.79
s-triazine CAS(12,9)	2^1^ *A*_1_	0.00	–0.02	0.00	0.00	0.59	–1.89	1.12	–0.27	1.68	–2.19
	3^1^ *A*_1_	0.00	0.04	0.00	0.00	0.15	1.08	0.32	0.56	0.51	1.68
	4^1^ *A*_1_	0.00	0.01	0.00	0.00	0.75	–1.05	1.66	0.27	2.42	–0.78
	1^1^ *A*_2_	0.00	–0.08	0.02	–0.04	0.34	0.07	0.25	0.13	0.53	0.08
	2^1^ *A*_2_	0.00	–0.07	0.02	–0.04	0.59	–0.27	0.46	0.08	0.99	–0.30
	3^1^ *A*_2_	0.00	–0.09	0.01	0.15	0.42	0.10	0.28	0.05	0.62	0.22
	1^1^ *B*_1_	0.00	0.05	0.00	0.00	–0.03	0.00	0.07	0.02	0.09	0.07
	1^1^ *B*_2_	0.00	–0.08	0.01	0.00	0.09	0.00	0.10	0.09	0.12	0.02
	MD	0.00	0.01	0.01	0.01	0.21	0.03	0.46	0.12	0.68	0.17
	MAD	0.00	0.04	0.01	0.01	0.21	0.30	0.46	0.26	0.69	0.53

In Table 5, for the s-triazine molecule, the ΔΔ*E* values of the VII component of NEVPTS produce significant errors in computing three ^1^ *A*_1_ excited states. The absolute ΔΔ*E* are larger than 1.0 eV for all the three ^1^ *A*_1_ states. To gain more insight into the large errors, again, the eigenvalues in the VII components of NEVPTS and AC0 in the ground and excited ^1^ *A*_1_ states are compared to the corresponding Koopmans eigenvalues in NEVPT2. The results are given in Figure 5. For all four states, the total number of eigenvalues in NEVPTS is similar to those in NEVPT2. For the ground state, the eigenvalues below 1.7 Hartree in NEVPTS match the Koopmans eigenvalues in NEVPT2 well. In contrast, for the three excited states, the eigenvalues obtained from the NEVPTS method exhibit lower energies compared to those derived from the NEVPT2 method. It is noteworthy that in the NEVPTS results for the three excited states, one or two negative eigenvalues are observed, which can potentially lead to intruder state problems.^89^ However, by omitting these negative eigenvalues in the NEVPTS calculations, the final correlation energies remain largely consistent with the original NEVPTS results. The inferior performance of NEVPTS in s-triazine is not due to these negative eigenvalues. The large errors of NEVPTS could be attributed to the imbalanced description of ground and excited states.

**Figure 5:**
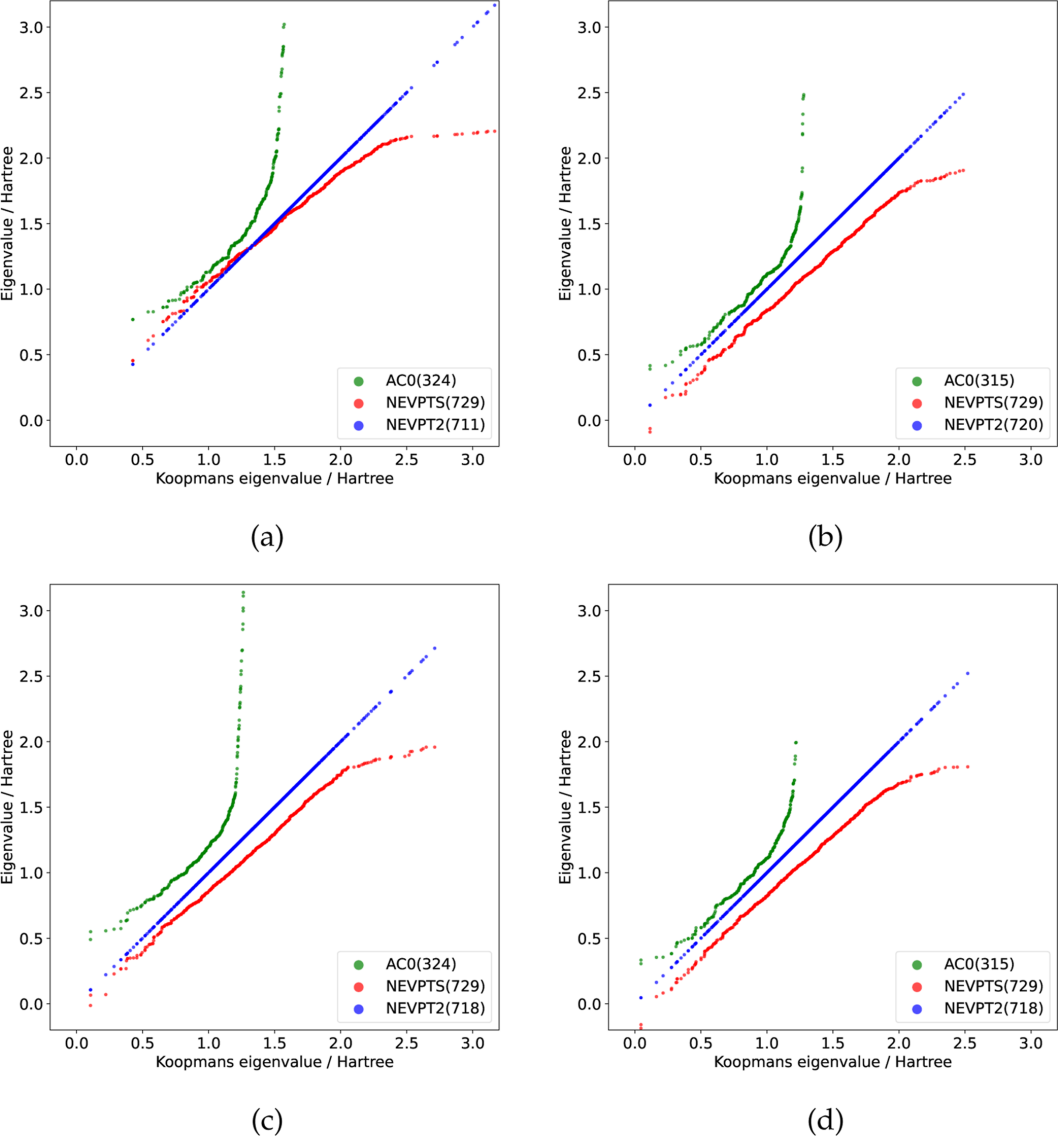
Eigenvalues (in Eh) appearing in the VII component of NEVPTS and AC0, compared with eigenvalues of Koopmans matrices in the VII subspace of NEVPT2 methods (denoted as “Koopmans eigenvalue” on the plot) for four states of s-triazine, (a) 1^1^ *A*_1_, (b) 2^1^ *A*_1_, (c) 3^1^ *A*_1_, (d) 4^1^ *A*_1_. The total number of eigenvalues in each method is given in parentheses.

## 4 Conclusions

In this work, we have developed a new MRPT method, NEVPTS. The wave function of NEVPTS is constructed using approximate singly excited CASSCF wave functions. Consequently, NEVPTS requires only 3rd-order RDMs, which makes NEVPTS computationally more efficient compared to NEVPT2. For the ground state PECs of the investigated dimers, NEVPTS achieves accuracy comparable with NEVPT2, surpassing that of AC0. The most significant improvement is observed for Cr_2_ dissociation curve. In the prediction of S-T gaps for biradicals, the NEVPTS performs comparably to NEVPT2, slightly outperforming AC0. However, for organic molecules, NEVPTS tends to overestimate excitation energies, with an accuracy comparable to AC0. Overall, the performance of NEVPTS is superior to AC0 but inferior to NEVPT2. However, its computational costs are lower than those of NEVPT2, particularly for calculations with large active spaces. Recently, some of us have developed the ff-AC0 method to enhance the accuracy of AC0,^99^ which may be applicable to improve the accuracy of NEVPTS. Efforts to enhance the accuracy of NEVPTS are ongoing and will be reported in subsequent studies. Additionally, the application of the NEVPTS method combined with selected CI references for large active space calculations will also be reported in forthcoming publications.

## Supplementary Material



## Appendix 1

### Tamm-Dancoff Approximation to AC0 (AC0tda)

In the theory of AC0,^93^ the excited states are constructed as

A.1
$$|\Psi_{\mu }^{ERPA}\rangle ={Ô}_{\mu }^{†}|\Psi_{0}\rangle$$

where

A.2
$${Ô}_{\mu }^{†}=\frac{1}{2}\sum_{r>s}\left({\left[X_{\mu }\right]}_{rs}{Ê}_{s}^{r}+{\left[Y_{\mu }\right]}_{rs}{Ê}_{r}^{s}\right)$$

For the adjoint excitation operator ${Ô}_{\mu }$ , the killer condition is assumed

A.3
$${Ô}_{\mu }|\Psi_{0}\rangle =0$$

Based on the Tamm-Dancoff approximation (TDA),^104^ an alternative excitation operator could be defined

A.4
$${\mathrm{Q̂}}_{\mu }^{†}=\frac{1}{2}\sum_{r>s}{\left[X_{\mu }\right]}_{rs}{Ê}_{s}^{r}$$

The excited states generated by ${\mathrm{Q̂}}_{\mu }^{†}$ are orthogonal to the ground state

$$\langle \Psi_{0}|\Psi_{\mu }^{TDA}\rangle =\left\langle \Psi_{0}\left|{\mathrm{Q̂}}_{\mu }^{†}\right|\Psi_{0}\right\rangle =0$$
A.5

It is trivial to prove that the excited states that satisfy the killer condition, also satisfy the orthogonal condition. However, this does not apply the other way around. Based on the ${\mathrm{Q̂}}_{\mu }^{†}$ operator, an alternative MRPT2 method could be defined as well, which could be named as AC0tda. In AC0tda, the excitation energies and the corresponding wave function are determined from variation equations obtained for the excitation energies

A.6
$$\langle \Psi_{0}|\left[{\mathrm{Q̂}}_{\mu },\left[{Ĥ}^{0},{\mathrm{Q̂}}_{\mu }^{†}\right]\right]|\Psi_{0}\rangle =w_{\mu }^{TDA}\left\langle \Psi_{0}\left|\left[{\mathrm{Q̂}}_{\mu },{\mathrm{Q̂}}_{\mu }^{†}\right]\right|\Psi_{0}\right\rangle$$

The matrix formulation of the variational equation reads

A.7
$$A^{0}X_{\mu }=w_{\mu }^{TDA}SX_{\mu }$$

The final correlation energy of AC0tda is computed by

A.8
$$E^{AC0tda}=\sum_{\lambda \mu }\left[\sum_{pqrs}\left(pq|rs\right){\left[SX_{\lambda }\right]}_{pq}{\left[SX_{\mu }\right]}_{rs}\right]\frac{X_{\lambda }B^{1}X_{\mu }}{w_{\lambda }+w_{\mu }}$$

and it differs from ERPA equations by the absence of the *B* ^0^ matrix.

If |Ψ_0_⟩ is the ground state, the orthogonal condition in the AC0tda method is equivalent to the killer condition in AC0, for some cases. Thus, the six components, I–V, and VIII″ are the same in AC0, NEVPTS and AC0tda. By comparing the working equations, one can see that the theory of NEVPTS is similar to that of AC0tda as well. The major difference is that no commutator is used to compute the single excitation wave function and final correlation energy in NEVPTS. Numerical tests have shown that AC0tda leads to much larger errors than AC0 for ground and excited states. Thus, it does not appear to be an attractive alternative.

## Appendix 2

### Comparison of Correlation Energy Expression of the VII Component (Subspace) in NEVPT2, NEVPTS and AC0

In NEVPT2, apart from the *V*_*ijab*_^PT2^ subspace, a generalized eigenvalue (GE) equation must be solved for each subspace

A.9
$$KC_{\mu }^{PT2}=\epsilon_{\mu }^{PT2}\Gamma C_{\mu }^{PT2}$$

for the II–VII subspaces, ***K*** and Γ are the various Koopmans ionization potential (IP) or electron affinity (EA) matrices and density matrices,^47^ respectively. For the VII subspace, *V*_*a*_^PT2^, the GE equation is given by

$$\langle \Psi_{0}|{Ê}_{a}^{\mathrm{t̅}}{Ê}_{\mathrm{v̅}}^{\mathrm{u̅}}[{Ĥ}^{act},{Ê}_{u}^{v}{Ê}_{t}^{a}]|\Psi_{0}\rangle C_{\mu }^{PT2}=\epsilon_{\mu }^{PT2}\left\langle \Psi_{0}\left|{Ê}_{a}^{\mathrm{t̅}}{Ê}_{\mathrm{v̅}}^{\mathrm{u̅}}{Ê}_{u}^{v}{Ê}_{t}^{a}\right|\Psi_{0}\right\rangle C_{\mu }^{PT2}$$
A.10

where ${Ĥ}^{act}$ is the active part of ${Ĥ}^{Dyall}$

A.11
$${Ĥ}^{act}=\sum_{tu}F_{tu}{Ê}_{u}^{t}+\frac{1}{2}\sum_{tuvw}\left(tu|vw\right){Ê}_{u}^{t}{Ê}_{w}^{v}$$

with *F*_pq_ being the generalized Fock matrix element. The final correlation energy of the *V*_*a*_^PT2^ subspace is computed as

A.12
$$E_{V_{a}}^{PT2}=-\sum_{\mu a}\frac{{\left|NC_{\mu }^{PT2}\right|}^{2}}{\epsilon_{\mu }^{PT2}+F_{aa}}$$

In eq A.12, only matrix elements *N*_uvta_ are required. In the absence of linear dependency in the IC wave functions, the total number of Koopmans eigenvalues of eq A.10 is *n* ^3^, where *n* is the number of active MOs.

In NEVPTS, the correlation energy of the *V*_ta,uv_^PTS^ component is computed as follows

$$E_{V_{ta,uv}}^{PTS}=\underset{tauv}{\sum }\;\underset{\lambda \mu }{\sum }{\left[{\mathrm{S̃}}^{act‐vir}{\mathrm{X̃}}_{\lambda }^{act‐vir}\right]}_{ta}\left(ta|uv\right){\left[{\mathrm{S̃}}^{act‐act}{\mathrm{X̃}}_{\mu }^{act‐act}\right]}_{uv}\frac{{\mathrm{X̃}}_{\lambda }^{act‐vir}N{\mathrm{X̃}}_{\mu }^{act‐act}}{{\mathrm{w̃}}_{\lambda }^{act‐vir}+{\mathrm{w̃}}_{\mu }^{act‐act}}=\underset{tauv}{\sum }\;\underset{\lambda \mu^{\prime }}{\sum }{\left[\Gamma^{1}C_{\lambda^{\prime }}^{IP1}\right]}_{t}\left(ta|uv\right){\left[{\mathrm{S̃}}^{act‐act}{\mathrm{X̃}}_{\mu }^{act‐act}\right]}_{uv}\frac{C_{\lambda^{\prime }}^{IP1}N{\mathrm{X̃}}_{\mu }^{act‐act}}{\epsilon_{\lambda^{\prime }}^{IP1}+F_{aa}+{\mathrm{w̃}}_{\mu }^{act‐act}}$$
A.13

here, $C_{\lambda^{\prime }}^{IP1}$ and $\epsilon_{\lambda^{\prime }}^{IP1}$ are the eigenpairs of the active part of CIS equation for the act-vir block

A.14
$$\langle \Psi_{0}|{Ê}_{a}^{\mathrm{t̅}}\left[{Ĥ}^{act},{Ê}_{t}^{a}\right]|\Psi_{0}\rangle C_{\lambda^{\prime }}^{IP1}=\epsilon_{\lambda^{\prime }}^{IP1}\left\langle \Psi_{0}\left|{Ê}_{a}^{\mathrm{t̅}}{Ê}_{t}^{a}\right|\Psi_{0}\right\rangle C_{\lambda^{\prime }}^{IP1},\left(\Gamma_{\mathrm{t̅}t}^{1}=\left\langle \Psi_{0}\left|{Ê}_{a}^{\mathrm{t̅}}{Ê}_{t}^{a}\right|\Psi_{0}\right\rangle \right)$$

the above equation is the same as the GE equation of *V*_*iab*_^PT2^ in NEVPT2.^100^ For the comparison of eigenvalues of the VII component in NEVPTS with respect to the Koopmans eigenvalues in NEVPT2, the term $\left(\epsilon_{\lambda^{\prime }}^{IP1}+{\mathrm{w̃}}_{\mu }^{act‐act}\right)$ is illustrated in Figures 4 and 5. If there is no linear dependency in solving the CIS equation (eq 20), the number of eigenvalues in the VII component of NEVPTS is also *n* ^3^.

The energy expression for the *V*_ta,uv_^AC0^ component is given by

$$E_{V_{ta,uv}}^{AC0}=\underset{tauv}{\sum }\underset{\lambda \mu }{\sum }{\left[\Gamma^{1}C_{\lambda^{\prime }}^{IP1}\right]}_{t}\left(ta|uv\right){\left[S^{act‐act}X_{\mu }^{act‐act}\right]}_{uv}\frac{C_{\lambda^{\prime }}^{IP1}\left(A^{1}+B^{1}\right){\left(Z^{+}\right)}_{\mu }^{act‐act}-C_{\lambda^{\prime }}^{AC0}\left(A^{1}-B^{1}\right){\left(Z^{-}\right)}_{\mu }^{act‐act}}{\epsilon_{\lambda^{\prime }}^{IP1}+F_{aa}+w_{\mu }^{act‐act}}$$
A.15

In this expression, *w*_μ_^act‑act^ is calculated from eq 5. The eigenvalues of the metric, $\left\langle \Psi_{0}\left|\left[{Ê}_{u}^{t},{Ê}_{v}^{w}\right]\right|\Psi_{0}\right\rangle$ , are additive inverse pairs, consisting of a positive-valued set and a corresponding negative-valued set. The negative-valued eigenpairs must be neglected in the subsequent ERPA eigenvalue problem. Thus, the maximum number of *w*_μ_^act‑act^ is $\frac{n\left(n-1\right)}{2}$ . Excluding the linear dependence in solving the ERPA equations, the number of eigenvalues in the VII component of AC0 is $\frac{n^{2}\left(n-1\right)}{2}$ , which is less than one-half of that in NEVPTS or NEVPT2.

## Notes

Absolute energies of N_2_, O_2_ and Cr_2_ molecules calculated using the NEVPTS, AC0, and NEVPT2 methods (PDF).
