# Supplementary material for: Approximation to Second Order N‑Electron Valence State Perturbation Theory: Limiting the Wave Function within Singles
Source: J Chem Theory Comput. 2025 May 28;21(11):5545–58. doi: 10.1021/acs.jctc.5c00582 (PMC12239084; doi:10.1021/acs.jctc.5c00582)
Supplement: Supplementary file 1 [file ct5c00582_si_001.pdf]

Supporting Information
Approximation to second order N-electron valence state perturbation theory: limiting the wave function within singles

Yang Guo*,† and Katarzyna Pernal‡

†*School of Chemistry and Chemical Engineering, Shandong University, Qingdao, Shandong 266237, China*

‡*Institute of Physics, Lodz University of Technology, ul. Wolczanska 217/221, 93-005 Lodz, Poland*

E-mail: yang.guo@sdu.edu.cn

Contents

1. Table S1. The absolute CASSCF, AC0, NEVPTS, and NEVPT2 energies of N_2 with CAS(10,8)/cc-pwCVQZ.

2. Table S2. The absolute CASSCF, AC0, NEVPTS, and NEVPT2 energies of O_2 with CAS(8,6)/cc-pwCVQZ.

3. Table S3. The absolute CASSCF, AC0, NEVPTS, and NEVPT2 energies of Cr_2 with CAS(12,12)/cc-pVQZ.

Table S1. The absolute CASSCF, AC0, NEVPTS, and NEVPT2 energies of N_2 with CAS(10,8)/cc-pwCVQZ.

N-N bond (Å)	CASSCF	AC0	NEVPTS	NEVPT2
1.0	-109.10366	-109.45831	-109.46192	-109.44722
1.1	-109.13981	-109.49045	-109.49491	-109.48069
1.2	-109.12079	-109.46852	-109.47392	-109.46008
1.3	-109.07663	-109.42246	-109.42886	-109.41533
1.4	-109.02391	-109.36869	-109.37608	-109.36278
1.5	-108.97159	-109.31605	-109.32425	-109.31107
1.6	-108.92440	-109.26912	-109.27766	-109.26452
1.7	-108.88484	-109.23021	-109.23840	-109.22516
1.8	-108.85418	-109.20012	-109.20709	-109.19372
1.9	-108.83252	-109.17836	-109.18356	-109.17011
2.0	-108.81862	-109.16346	-109.16698	-109.15349
2.1	-108.81041	-109.15367	-109.15596	-109.14241
2.2	-108.80582	-109.14740	-109.14889	-109.13527
2.3	-108.80335	-109.14343	-109.14443	-109.13074
2.4	-108.80205	-109.14091	-109.14157	-109.12784
2.5	-108.80136	-109.13928	-109.13975	-109.12595
2.6	-108.80100	-109.13820	-109.13858	-109.12469

Table S2. The absolute CASSCF, AC0, NEVPTS, and NEVPT2 energies of O_2 with CAS(8,6)/cc-pwCVQZ.

O-O bond (Å)	CASSCF	AC0	NEVPTS	NEVPT2
1.0	-149.65776	-150.16138	-150.16271	-150.15529
1.1	-149.74087	-150.24034	-150.24241	-150.23627
1.2	-149.76328	-150.25866	-150.26181	-150.25672
1.3	-149.75571	-150.24689	-150.25134	-150.24703
1.4	-149.73465	-150.22137	-150.22716	-150.22334
1.5	-149.70895	-150.19085	-150.19779	-150.19419
1.6	-149.68343	-150.16011	-150.16781	-150.16422
1.7	-149.66085	-150.13181	-150.13981	-150.13612
1.8	-149.64275	-150.10761	-150.11539	-150.11159
1.9	-149.62990	-150.08861	-150.09569	-150.09188
2.0	-149.62217	-150.07533	-150.08145	-150.07775
2.1	-149.61841	-150.06717	-150.07258	-150.06898
2.2	-149.61701	-150.06289	-150.06776	-150.06422
2.3	-149.61670	-150.06068	-150.06532	-150.06179
2.4	-149.61679	-150.05957	-150.06411	-150.06057
2.5	-149.61699	-150.05900	-150.06347	-150.05992
2.6	-149.61719	-150.05869	-150.06312	-150.05955

Table S3. The absolute CASSCF, AC0, NEVPTS, and NEVPT2 energies of Cr_2 with CAS(12,12)/cc-pVQZ.

Cr-Cr bond (Å)	CASSCF	AC0	NEVPTS	NEVPT2
1.3	-2086.44475	-2087.48962	-2087.50491	-2087.49422
1.4	-2086.57647	-2087.62044	-2087.63977	-2087.62828
1.5	-2086.63412	-2087.67863	-2087.70069	-2087.68864
1.6	-2086.65413	-2087.69766	-2087.72220	-2087.70978
1.7	-2086.65890	-2087.69917	-2087.72385	-2087.71128
1.8	-2086.66101	-2087.69448	-2087.71648	-2087.70412
1.9	-2086.66517	-2087.68971	-2087.70741	-2087.69529
2.0	-2086.67168	-2087.68707	-2087.70030	-2087.68829
2.1	-2086.67949	-2087.68673	-2087.69627	-2087.68426
2.2	-2086.68737	-2087.68788	-2087.69483	-2087.68273
2.3	-2086.69454	-2087.68950	-2087.69478	-2087.68256
2.4	-2086.70062	-2087.69094	-2087.69514	-2087.68282
2.5	-2086.70553	-2087.69202	-2087.69529	-2087.68295
2.6	-2086.70934	-2087.69236	-2087.69502	-2087.68271
2.7	-2086.71219	-2087.69202	-2087.69428	-2087.68202
2.8	-2086.71424	-2087.69116	-2087.69308	-2087.68096
2.9	-2086.71564	-2087.68987	-2087.69158	-2087.67956
3.0	-2086.71653	-2087.68828	-2087.68981	-2087.67795
